# Comparison of Effectiveness and Selectiveness of Baited Traps for the Capture of the Invasive Hornet *Vespa velutina*

**DOI:** 10.3390/ani14010129

**Published:** 2023-12-29

**Authors:** Sandra V. Rojas-Nossa, Salustiano Mato, Pilar Feijoo, Aarón Lagoa, Josefina Garrido

**Affiliations:** Department of Ecology and Animal Biology, University of Vigo, 36310 Vigo, Pontevedra, Spain; smato@uvigo.gal (S.M.); mfeijoo@uvigo.gal (P.F.); aaron.lagoa@uvigo.gal (A.L.);

**Keywords:** Asian hornet, control method, impacts, invasive species, traps, *Vespa velutina*, vespid

## Abstract

**Simple Summary:**

Baited traps are commonly used to reduce the invasive population of the Asian hornet *Vespa velutina*. However, the method is controversial because the evidence suggests that spring trapping is not effective in reducing the population and because of the high number of bycatches. However, new models have appeared in the market claiming selectiveness. The objective of this study was to compare the effectiveness and selectiveness of four types of traps in a coastal region of northeastern Spain. The Eva (E) trap had a higher effectiveness and a higher selectiveness. The prototype (R trap) had the lowest effectiveness and selectiveness. The VespaCatch (V) and Econex (X) traps had higher capture rates, but also the lowest selectiveness, with captures of vulnerable native species. The results indicate that, with the tested traps, bait trapping continues to be environmentally unsustainable and not recommended as a control method in regions with an established invasive population.

**Abstract:**

The invasive hornet *Vespa velutina* affects apiculture, biodiversity, and human health. The use of baited traps with the aim of reducing the population and mitigating its impacts is a common practice. However, the lack of impact on the number of colonies and the high capture rate of non-target insects make it controversial. Our objective was to compare the effectiveness and selectiveness of four trap models. We measured effectiveness as the capture rate of *V. velutina* per day per trap, and selectiveness as the capture rate of *V. velutina*/capture rate of non-target species. The E trap had better performance with a higher selectiveness and effectiveness. Traps V and X had a higher effectiveness but the lowest selectiveness, with high capture rates of native insects, including threatened species. The R trap had the lowest effectiveness and selectiveness. Results show that small changes in the design can contribute to achieving more sustainable tools. Otherwise, with the current tools, bait trapping continues to be non-sustainable from an environmental perspective due to the impacts on native biodiversity.

## 1. Introduction

Invasive hornets cause impacts on ecosystems through predation and competition for resources [1]. Besides direct effects, some control methods indirectly affect non-target species, increasing the impacts of invasive species on native biodiversity [2]. Aiming at the highest effectiveness and selectiveness of tools is of capital importance for developing sustainable management strategies.

Currently, Europe is experiencing a significant increase in the populations of some invasive alien species (i.e., those whose presence in a region is attributable to human actions, accidental or deliberate, with detrimental effects on economy, biodiversity, or health [3]). Also, there is a rising of neonative species [4]. Eusocial hymenopterans of the family Vespidae are prone to arriving, expanding, and invading into new regions. Some examples of invasive species are *Vespa velutina* Lepeletier 1836 and *Vespa bicolor* Fabricius 1787, which were accidentally introduced from Asia [5,6]. Differently, *Vespa orientalis* Linnaeus 1771, historically had a distribution limited to the eastern Mediterranean, but it is currently expanding towards western and northern regions, probably, facilitated by human transport and/or climate change [7]. Among these, the most remarkable case is *V. velutina*. It was first detected in France in 2004, and since then, the population spread and increased, becoming an invasive alien species of Union concern according to European regulations (European Commission implementing regulation (EU) 2016/1141). The species has also successfully invaded some Asian countries, including South Korea and Japan [8,9]. Its success as an invasive species is related to its ability to establish new populations from the minimum propagule (one mated single queen), its high reproductive rate, and its versatile generalist behavior [10], among others. 

*Vespa velutina* adults consume carbohydrates and obtain protein by scavenging and predating arthropods as a food resource for the brood [11]. In the invaded regions, the predation pressure affects native entomofauna, in particular insect pollinators, hunted in places where they congregate, such as blooming plants or the entrance of colonies of social hymenopterans [1,12,13]. In particular, these hornets are skilled hunters of honey bees [5]. They specialize in hawking workers returning to the hive by waiting in hovering flight near the entrance until they catch one prey. The predation pressure of *V. velutina* on *A. mellifera* colonies triggers a phenomenon known as the *paralysis of the colony* [14,15]. The Western honey bee (*Apis mellifera* Linnaeus 1758) is a species native to Europe, mostly managed, and its survivorship is the basis for the beekeeping sector and provides crucial pollination services for crops and wild plants [16]. As a consequence of the paralysis, there is an increase of 30–50% in colony death [15], with an estimated economic impact for the beekeeping sector of €30.8 million per year in France, related to colony replacement [17]. Moreover, when hunting and feeding on nectar in flower patches, the invasive hornets change the frequency and the behavior of pollinators through predation and competition, which ultimately affects the reproduction of plants [1,12]. Added to these impacts, the defensive behavior of *V. velutina* and its powerful venomous sting represents a new threat to human beings, with an increase in the burden on healthcare systems [18].

Different sectors mobilize efforts to prevent, eradicate, and develop control strategies in the regions invaded by *V. velutina* [19,20]. However, the only documented case of eradication from a territory occurred during the period of establishment in the Mediterranean island of Mallorca [21]. Its success was due to a rapid action strategy that included a combination of several methods, such as early detection and coordinated joint work between scientists, the public sector, technicians, and citizens. This, together with a significant distance to the mainland, reduced the chances of re-introduction. Other cases demonstrate that once established in a region, the control tasks for containment and long-term management are expensive, challenging, and often unsuccessful [22]. This is related to the low efficiency of current tools to reduce the population [19]. Some of them are effective but their effect is local, such as the muzzles or the electric harps, which help to reduce the negative impacts for honey bees in apiaries [23,24]. Nest destruction is one of the current alternatives to decrease the threats to biodiversity and the risks for people. There are advances in the methods to detect and destroy nests [25,26,27], but these involve important efforts and resources. In France, the estimated cost for nest destruction was € 23 million from 2006 to 2015 [28].

The use of baited traps is a practice aimed at reducing the population of *V. velutina*. However, these traps capture and kill high quantities of individuals of non-target taxa, regardless of the trap model, the bait, the season, or the region (see, for example, [23,29,30,31]). It is argued that these bycatches are justified because some hornet queens are captured, and this prevents the founding of colonies that otherwise could consume several thousands of insects each season. However, this argument lacks support because many insect groups captured in traps are not the same as those consumed by the invasive species, thus adding pressure on native biodiversity [23]. Considering the dependence of food security on the services provided by insects together with the accelerated declines of their populations and their consequent cascade effects observed in the last decades, the scientific community has called for immediate action to stop the drivers of insect extinctions [32,33].

This study aimed to compare the performance of four types of traps, assessed as the capture rate of *V. velutina* (effectiveness) and the relative capture of non-target insects (selectiveness). Three of these models were commercial traps, for which the associated information in the prospect claimed either to be particularly designed for the capture of *V. velutina*, or to be selective for the capture of the species. The fourth model was a prototype designed and recently patented by a beekeeper who was positive about the performance and selectiveness of his invention.

## 2. Materials and Methods

### 2.1. Field Work

**Figure 1:**
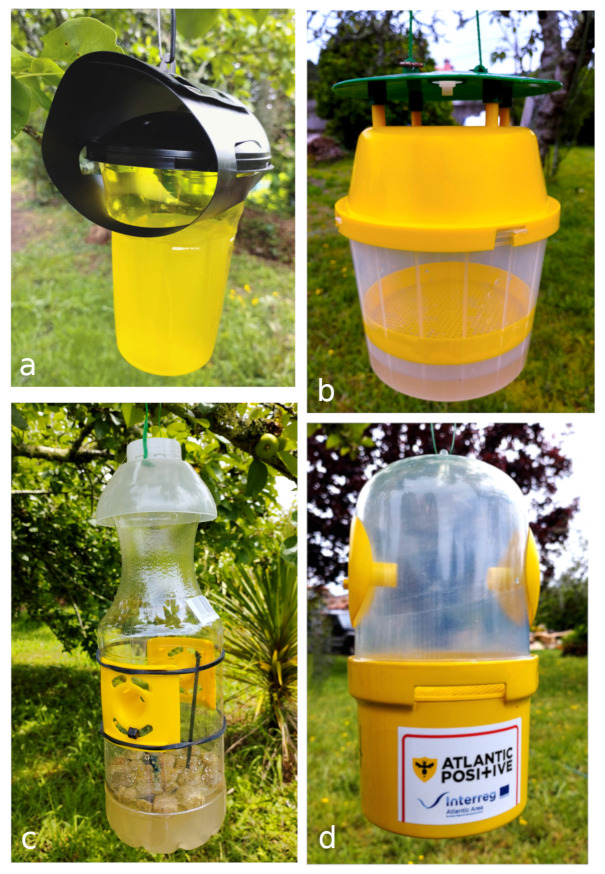
The effectiveness and selectiveness of four trap models were tested: (**a**) VespaCatch (V trap), (**b**) Econex (X trap), (**c**) Eva (E trap), and (**d**) a trap in development (R trap). All traps were filled with the same type and quantity of bait.

The sampling period lasted from 19 April to 15 July 2022. We tested four types of traps (three commercial and one prototype). The VespaCatch^®^ trap (V trap onwards), Véto-pharma, was sourced from Mel Niño do Corvo, O Rosal (Spain). The V trap has a black lid with two lateral entrance holes and a yellow plastic container that holds the liquid bait, where the insects die by drowning (Figure 1a). The Econex^®^ trap (X trap onwards), Econex, was sourced from Mel Niño do Corvo, O Rosal (Spain). The X trap has a green roof, a yellow lid with a central hole, and a transparent container which is divided by a plastic mesh in two parts, the base of which contains the liquid bait and a main chamber where insects die by exhaustion (Figure 1b). The Eva^®^ trap (E trap onwards), was sourced from Eva, Oviedo (Spain). The trap E has a transparent plastic bottle with two lateral yellow pieces. Each of them has one bigger hole used as an entrance and four smaller holes used as an exit for smaller insects. The trap is provided with expanded clay balls to avoid insect drowning and plastic sticks to allow them to crawl towards the exits (Figure 1c). The insects unable to escape die by exhaustion and/or drowning. The fourth trap was a prototype in the phase of industrial development (R trap onwards. Patent ES1285161U), which has a transparent dome-shaped lid provided with two lateral entrances and a yellow container that holds the liquid bait (Figure 1d). The container is divided by a plastic mesh from the main chamber where insects die by exhaustion. 

**Figure 2:**
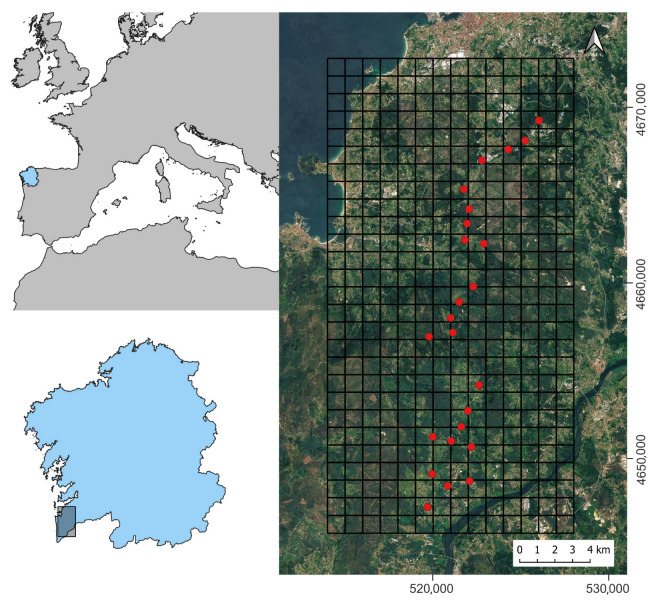
Location of the 24 baited-traps (red dots) used in the study in the southwest of the Pontevedra province, Galicia, Spain. UTM coordinates (zone 29T).

We set six traps for each model, making a total of 24 traps (4 models × 6 traps per model = 24 traps). To set traps in the field, we divided the terrain into a 1 km^2^ grid in a coastal region, of southwest Pontevedra, Spain. Only grids with a relative abundance of *V. velutina* colonies the previous year between 1–8 nests/km^2^ in 2020 were chosen (unpublished data, Xunta de Galicia). Each trap was randomly assigned to one sector and was left active during the whole study. The minimum distance between neighboring traps was 1 km (Figure 2).

To compare the performance of these traps, they were filled with 300 mL of the same liquid attractant as bait. The bait was prepared with the following proportions: 1 L water, 400 g sugar, and 10 g of bakery yeast. The ingredients were homogenously mixed two days before setting the trap for the first time or before renewing the bait after collecting the samples. This liquid attractant has been used by Galician beekeepers with success in recent years (N. and R. González, Pers. Comm.). The traps were hung at a height of 1.5 m, from tree branches facing south, so they had similar conditions of luminosity. The captured insects were collected approximately every two weeks (14.08 ± 2.35 days), transferred in 70% ethanol, and the bait was renewed. Each sample collection batch was considered one sampling for the data analysis. Therefore, we had a total of 6 samplings: one in the second half of April, two in May and June (corresponding to the first and the second half of these months), and one in the first half of July. Despite being labeled as scientific material, one R trap was lost, leaving a gap of two samples while it was replaced. Each sample was labeled in the field. In the laboratory, the label was replaced by a numerical code and then analyzed. With this procedure, we ensured that the person who performed the sample analysis was blinded to the treatment associated with each sample, reducing the chances of biases [34]. The insects were identified and counted to the order level (for Diptera, Coleoptera, and Lepidoptera), family (within Hymenoptera), or species in the case of *Vespa velutina* and *Vespa crabro* L. 1758. 

### 2.2. Statistical Analyses

The capture rate of *V. velutina* and other insect groups was calculated as the number of individuals per trap per day. The effectiveness was measured for each sample as the capture rate of *V. velutina*, while the selectiveness was calculated for each sample as the ratio between the capture rate of *V. velutina* per trap per day/capture rate of non-target insects per trap per day. The selectiveness was log-transformed. To compare the differences between the effectiveness and selectiveness of traps, we fitted generalized linear mixed models (GLMMs) with Poisson distribution. The models were fitted with the package *lme4* for R software [35]. To compare the differences between the capture rates of Lepidoptera, native Vespidae, and Coleoptera, we fitted GLMMs with negative binomial distribution. The models were fitted and tested for overdispersion with the packages *glmTMB* [36] and *DHARMa* [37] for R software. The type of trap and sampling and its interaction were included as fixed terms. To control for pseudoreplication, the identity of the traps was included as a random factor into the model. Overdispersion was not an issue. Post hoc pairwise tests were performed with the package *lsmeans* [38]. 

## 3. Results

### 3.1. Effectiveness and Selectiveness of Traps

**Figure 3:**
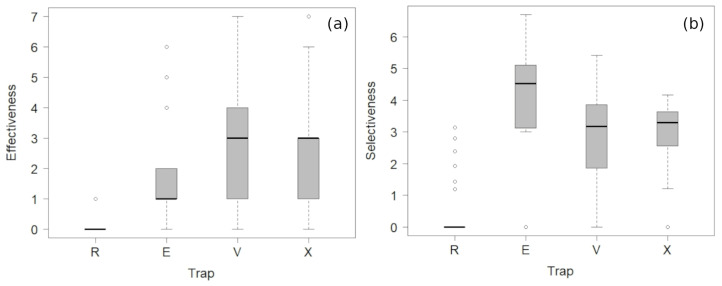
Effectiveness and selectiveness of baited traps aimed to capture the invasive Asian hornet *Vespa velutina*. (**a**) Effectiveness (capture rate of *V. velutina* per day per trap). (**b**) Log-transformed selectiveness (capture rate of *V. velutina*/capture rate of non-target insects).

The effectiveness for capturing *V. velutina* was significantly different among traps (χ^2^ = 40.85; df = 3; *p* < 0.001) and season (χ^2^ = 38.34; df = 5; *p* < 0.001), but not for the interaction between the type of the trap and the season (χ^2^ = 9.39; df = 15; *p* = 0.856). Trap R had the lowest effectiveness across the study, with significant differences in comparison with the effectiveness of traps E, V, and X (Figure 3a and Table S1 in Supplementary material). The capture rate of *V. velutina* was lower in the second half of April for all traps, with an increasing tendency until the first half of June (Table 1). During the second half of June, the capture rate suffered a decrease and afterward had the tendency to increase in traps E and X. 

All traps captured different groups of non-target insects (Table 2). The type of trap, the sampling, and the interaction between trap and sampling affected selectiveness (χ^2^ = 155.65; df = 3; *p* < 0.001 for traps, χ^2^ = 113.36; df = 5; *p* < 0.001 for samplings and χ^2^ = 75.17; df = 15; *p* < 0.001 for the interaction). Overall, the trap E had the highest selectiveness (Figure 3b, Figure S1 and Table S3 in Supplementary Material). When comparing the selectiveness across the study, the trap E had a higher selectiveness than X and V in May, and in the first half of June and July in comparison with trap X. The R trap had the lowest selectiveness. The selectiveness was lower for the traps V and X, and did not differ significantly between them. 

### 3.2. Capture of Non-Target Taxa

Diptera was the most captured group, followed by Formicidae and Coleoptera (Table 2). Overall, traps R, X, and V captured higher proportions of non-target taxa. It is worth noting the high capture rate of Lepidoptera in the X trap (χ^2^ = 95.14; df = 3; *p* < 0.001 for traps, χ^2^ = 69.98; df = 5; *p* < 0.001 for samplings and χ^2^ = 90.32; df = 15; *p* < 0.001 for the interaction trap and sampling, and Table S4 in Supplementary material), and native Vespidae, including *V. crabro,* in the V trap (χ^2^ = 33.96; df = 3; *p* < 0.001 for traps, χ^2^ = 139.73; df = 5; *p* < 0.001 for samplings and χ^2^ = 118.48; df = 15; *p* < 0.001 for the interaction trap and sampling, and Table S5 in Supplementary material). Traps X and V also had a high capture rate of Coleoptera (χ^2^ = 58.36; df = 3; *p* < 0.001 for traps, χ^2^ = 43.28; df = 5; *p* < 0.001 for samplings and χ^2^ = 68.28; df = 15; *p* < 0.001 for the interaction trap and sampling, and Table S6 in Supplementary material). The capture of *Lucanus cervus* (L. 1758) in the X trap was particularly noteworthy.

## 4. Discussion

This work explores the mechanical typology of traps for the capture of the invasive hornet *V. velutina* (effectiveness), and the relative importance of the capture of the target species in relation to the capture of other non-target insects (selectiveness). 

All studied traps were able to capture *V. velutina* to some extent, but also other non-target insects. Trap R had the lowest effectiveness and selectiveness. This trap captured a high amount of non-target insects, particularly small Diptera and Formicidae, which were able to enter through the plastic mesh into the chamber that contains the liquid bait. The traps V and X had the highest effectiveness among tested traps, but also a lower selectiveness in comparison with trap E. The captured insects included taxonomic groups that are not usually predated by *V. velutina*, such as Lepidoptera or Coleoptera [39]. Particularly worrying is the capture of vulnerable species, such as the European stag beetle (*L. cervus*), included in the IUCN Red List listed as Near Threatened [40]. The trap X has the biggest entrance of all tested models, which is probably one of the reasons that allowed this species to be trapped. In addition, the European hornet (*V. crabro*) was commonly captured in the V trap and to a lesser extent in the X and E traps. The species is protected by European regional regulations aimed at protecting native biodiversity, and it has a special category of protection in some European countries [41]. Similar results were obtained in another study that evaluated the same response (effectiveness and selectiveness) but of a factorial combination of three traps and three liquid baits [23]. In that case, the researchers tested traps in which insects die by drowning, different from some of the traps evaluated here, in which insects die by exhaustion. This suggests that preventing insects from drowning is not the only measure that reduces the killing of non-target insects. In fact, the X trap (in which insects die by exhaustion) was originally a trap intended to capture Lepidopteran pests and is now commercialized for the capture of the invasive hornet without any modification. As a result, this trap has a particularly high capture rate of Lepidoptera, which is 4.3 times higher than the capture of Lepidoptera by the V trap and 10 times higher than the capture rate of the E trap. The capture rate of Coleopterans in the X trap was also 2.3 times higher than the capture rate for the V trap and 7.5 times higher than the capture rate for the E trap. Because Lepidopterans and Coleopterans are known as some of the most important groups of pollinators for wild and cultivated plants, and thus for food security [32], the potential impact of the use of baited traps over these groups is unwanted. The evidence provided here supports a previous study about the quantity and diversity of preyed insects by *V. velutina* colonies in Europe, which suggested that four to six small baited traps can catch as many insects as one *V. velutina* colony can prey upon [39]. Besides the loss of biomass, biodiversity, and ecosystem services, terrestrial arthropods are fundamental for ecosystem functioning [42]. Thus, the decline of insect populations is associated with co-extinctions and bottom-up and top-down extinction cascades [43]. Because it is a global concern to implement urgent actions to reduce our impacts on biodiversity [33], we encourage companies and users to prioritize the ecological sustainability of tools used to control the populations of invasive species. 

Trap E achieved a higher selectiveness in comparison with other traps. Its design likely contributed to the reduction of bycatches. These include the position and size of the entrance holes, which exclude bigger animals from entering the trap, added to the presence of a system that allows smaller insects to escape. Although it would be necessary to test the effect of single traits on the performance of the whole trap, our results support previous evidence that suggests the relevance of the design as one determinant factor related to the performance of the traps [23]. It is also worth noting that the lateral openings of the trap E caused a more rapid drying of the bait, particularly on warmer days. This suggests that the overall effectiveness of the trap could be improved by renewing the bait more often than the schedule used in the study (two weeks), increasing the maintenance required for the trap.

Except for the E trap, the proportion of *V. velutina* captured in the tested traps was similar to the proportion observed in other studies made in different regions, with different time after establishment and biogeographic conditions (Table 3). In all cases, the percentage of *V. velutina* captured in comparison with other species was low to very low.

During the invasion process of *V. velutina*, baited traps have been used with different purposes, such as a sampling method for testing scientific hypotheses or as part of surveillance and control strategies against the invasive species. In the first case, for example, these have been used to assess the abundance of the invasive population of *V. velutina* across seasons and/or sites within regions [1,13,46], study the environmental conditions associated with the invasion [30], or assess causal effects over native populations [47]. In the second case, these have been successfully employed in order to detect the arrival of the species into new regions [48], to monitor the expansion of or decline in the population [21,49], or as a part of the eradication strategy during the establishing period in a Mediterranean island [21]. Besides these, the most common use of baited traps, either commercial or homemade, is aimed at reducing the number of adult *V. velutina* in the environment. Spring trapping of queens near apiaries is a common measure practiced by beekeepers and included in management plans to reduce the number of nests [50]. However, the evidence suggests that, once the species is established, spring trapping is an inefficient method to manage the population of *V. velutina* because it does not influence the number of nests [22]. This is related to the low number of captured foundresses in comparison with the number of queens produced by the colonies in the same area during the former reproductive season, and the enormous colonization capacity of the species [44,51].

The significant changes in the capture rates of *V. velutina* reflect an increasing tendency throughout spring, followed by a decline at the beginning of summer, and an increase after July. This temporal pattern could reflect the period of queen activity in the field until the first half of June, then a subsequent period of fewer captures corresponding to the period of transition between the queen colony phase and the emergence of workers [5]. This is later followed by the growth and maturation of the colony. Further studies dealing with the capture rate of different castes in response to the annual cycle, and its interaction according to the particular environmental conditions for each region would be necessary to improve detection and early control actions aimed at prevention and eradication strategies. Moreover, studies identifying captured taxa at the species level can provide further information about the impact of the traps on endangered native species.

Here, we compared the relative performance of different trap models in terms of capture rates of *V. velutina* and other insects, but not the efficiency of the traps in achieving the objective of reducing the population. However, according to our data, the effectiveness (with means of 0.01, 1.27, 2.19, and 2.27 *V. velutina* per trap per day, for traps R, E, X, and V, respectively) is disproportionately low compared to the number of new foundresses that can be produced each reproductive season in the region of the study (approx. 350 gynes per colony [5], and an estimated number of 14–17 colonies per km^2^ [52]). We also found that for each *V. velutina* captured in spring, these traps killed a mean of 80.15 (R trap), 9.98 (E trap), 97.31 (V trap), and 64.61 (X trap) individuals of non-target species. Therefore, our results, together with those in the literature, indicate that, with the current tools, bait trapping is environmentally unsustainable and not recommended as a control method in regions with an established invasive population.

## 5. Conclusions

The tested traps differed in terms of the capture rate of *V. velutina* (effectiveness) and other insects (selectiveness). The trap E is an improvement in terms of selectiveness when comparing the capture rates of other traps, suggesting that details in the design of the traps can significantly reduce bycatches. High-performance baited traps, namely having both high effectiveness and high selectiveness, have not been developed and tested so far. This, together with the lack of evidence of the impact of trapping on the invasive population, indicates that bait trapping is an added threat to native biodiversity.

## Figures and Tables

**Table 1:** Captured *Vespa velutina* per trap in four types of traps tested in spring and early summer. Samples were collected in the first (I) or the second (II) half of each month. The tested traps were abbreviated as prototype (R), Eva^®^ (E), VespaCatch^®^ (V), and Econex^®^ (X). Reported numbers correspond to the mean of individuals captured per sample ± standard deviation.

		Trap		
Sampling	R	E	V	X
1 (April—II)	0	0	0.16 ± 0.30	0.11 ± 0.10
2 (May—I)	0	0.52 ± 0.21	1.33 ± 1.31	1.84 ± 0.95
3 (May—II)	0	1.25 ± 0.79	2.13 ± 1.44	2.29 ± 0.51
4 (June—I)	0.01 ± 0.03	2.35 ± 1.74	4.43 ± 1.52	3.19 ± 1.84
5 (June—II)	0.02 ± 0.05	0.17 ± 0.41	3.38 ± 1.34	2.32 ± 1.12
6 (July—I)	0.02 ± 0.03	2.62 ± 2.07	2.18 ± 0.86	3.36 ± 2.14

**Table 2:** Captured insects in four types of baited traps aimed to capture *Vespa velutina*. Reported numbers correspond to the Mean of individuals captured per sampling per trap ± standard deviation. Non-target/*V. velutina* = mean number of individuals of non-target species captured per each *V. velutina* captured. The overall proportion represents the number of individuals of non-target species captured per each *V. velutina* trapped throughout the study (calculated as the sum of all non-target individuals/sum of all *V. velutina* individuals for each type of trap). The tested traps were abbreviated as VespaCatch® trap (V), Econex® trap (X), Eva® trap (E) and prototype (R).

	Trap			
Captured Insects	R	E	V	X
All insects	81.62 ± 80.28	136.81 ± 147.47	1501.86 ± 1352.99	1643.58 ± 2326.26
Non-target insects	76.89 ± 80.06	118.86 ± 132.81	1485.83 ± 1350.86	1612.11 ± 2309.68
Non-target/ *V. velutina*	80.15 ± 80.38	9.98 ± 11.78	97.31 ± 112.69	64.61 ± 63.32
Vespidae				
*Vespa velutina*	0.21 ± 0.48	17.94 ± 19.80	32.06 ± 24.41	31.47 ± 24.55
*Vespa crabro*	0	0.19 ± 0.40	1.74 ± 2.39	0.97 ± 1.79
Other vespids	0	4.36 ± 6.29	16.83 ± 20.11	9.00 ± 10.65
Diptera	49.77 ± 63.37	91.06 ± 117.96	1273.64 ± 1314.54	1350.64 ± 2267.52
Formicidae	26.27 ± 137.31	12.75 ± 39.46	137.31 ± 260.41	156.56 ± 444.07
Coleoptera	5.26 ± 7.37	5.67 ± 15.72	18.31 ± 31.40	42.47 ± 36.91
Lepidoptera	0.03 ± 0.17	4.67 ± 5.79	19.72 ± 22.13	46.08 ± 36.42
Anthophila	0	0.14 ± 0.35	0.39 ± 0.60	0.80 ± 2.01
Other groups	0.09 ± 0.52	0.03 ± 0.17	1.92 ± 5.45	4.86 ± 16.96
Overall	395.4	6.6	45.9	51.2

**Table 3:** Proportion of *Vespa velutina* of the total number of insects captured in studies performed in different countries and seasons.

Country Type of Trap	Season	% of *V. velutina*	Year of Sampling [Reference]
Spain			[This study]
Overall		2.43	
R	Spring	0.19	
E	Spring	16.21	
V	Spring	3.31	
X	Spring	2.50	
France			
Funnel trap	Spring	1.70	2011 [44]
Spain			
Avispa’clac, VétoPharma, and home-made trap	Spring	0.9	2016 [23]
Italy			
PET bottle with TapTrap and VespaCatch	Spring-autumn	1.02	2018 [31]
Spain			
VespaCatch	All seasons	2.23	2020–2021 [45]

## Data Availability

The dataset generated during the current study are available in the Figshare repository. https://doi.org/10.6084/m9.figshare.23502354.

## Supplementary Materials

The following supporting information can be downloaded at: https://www.mdpi.com/article/10.3390/ani14010129/s1, Tables S1 and S2: Post-hoc tests for Effectiveness, Figure S1: Selectiveness of the four types of baited traps tested throughout the study, Table S3: Post-hoc tests for Selectiveness, Table S4: Post-hoc tests for the capture rate of Lepidoptera, Table S5: Post-hoc tests for the capture rate of native Vespidae, and Table S6 Post-hoc tests for the capture rate of Coleoptera.
