# Supplementary material for: Comparison of Effectiveness and Selectiveness of Baited Traps for the Capture of the Invasive Hornet Vespa velutina
Source: Animals (Basel). 2023 Dec 29;14(1):129. doi: 10.3390/ani14010129 (PMC10778013; doi:10.3390/ani14010129)
Supplement: Supplementary file 1 [file animals-14-00129-s001.zip › animals-2752552-supplementary.pdf]

SUPPLEMENTARY MATERIAL

Article

Comparison of effectiveness and selectiveness of baited traps for the capture of the invasive hornet *Vespa velutina*

Sandra V. Rojas-Nossa*, Salustiano Mato, Pilar Feijoo, Aarón Lagoa and Josefina Garrido

*Correspondence: *sarojas@uvigo.gal*

Table S1. Post-hoc tests for Effectiveness. Least-squares means for the fixed factor **Trap** of the GLMM model: **Effectiveness** ~ Trap * Sampling + (1 ǀ trap_ID). Pairwise significantly differences ($p \leq 0.05$) in **bold**. The tested traps were abbreviated as VespaCatch® trap (V), Econex® trap (X), Eva® trap (E) and prototype (R).

Sampling	Trap	Estimate	p-value
II-April	R - E	-0.974	1.0000
	R - V	-0.203	1.0000
	R - X	-0.206	1.0000
	E - V	-0.193	1.0000
	E - X	-0.196	1.0000
	V - X	-0.288	0.9819
I-May	R - E	-0.210	1.0000
	R - V	-0.215	1.0000
	R - X	-0.218	1.0000
	E - V	-0.511	0.7569
	E - X	-0.773	0.4000
	V - X	-0.262	0.9251
II-My	R - E	-1.26	0.3998
	R - V	-1.90	0.0560
	R - X	-1.90	0.0560
	E - V	-0.642	0.4910
	E - X	-0.642	0.4911
	V - X	0.00002	1.0000
I-June	R - E	-2.82	**0.0329**
	R - V	-3.40	**0.0046**
	R - X	-3.09	**0.0135**
	E - V	-0.580	0.2810
	E - X	-0.270	0.8606
	V - X	0.310	0.6905
II-June	R - E	-2.26	0.1441
	R - V	-3.18	**0.0101**
	R - X	-2.83	**0.0303**
	E - V	-0.918	0.1122
	E - X	-0.573	0.5418
	V - X	0.345	0.7002
I-July	R - E	-2.32	**0.0104**
	R - V	-2.08	**0.0288**
	R - X	-2.44	**0.0052**
	E - V	0.242	0.8999
	E - X	-0.120	0.9821
	V - X	-0.363	0.6837

Table S2. Post-hoc tests for Effectiveness. Least-squares means for the fixed factor **Sampling** of the GLMM model: Effectiveness ~ Trap * Sampling + (1 | trap_ID). Pairwise significantly differences ($p \leq 0.05$) in **bold**. The samplings were made approximately every two weeks (14.08 ± 2.35 days), from 19 April to 15 July 2022. Thus, each month was divided into two samplings and abbreviated as follows: 2nd half of April (II-Ap), 1st half of May (I-My), 2nd half of May (II-My), 1st half of June (I-Jn), 2nd half of June (II-Jn) and 1st half of July (I-Jl).

Trap	Samplings	Estimate	p-value
Prototype	(I-Jl) - (I-Jn)	0.694	0.9932
	(I-Jl) - (I-My)	0.199	1.0000
	(I-Jl) - (II-Ap)	0.199	1.0000
	(I-Jl) - (II-Jn)	0.693	0.9932
	(I-Jl) - (II-My)	-0.182	1.0000
	(I-Jn) - (I-My)	0.192	1.0000
	(I-Jn) - (II-Ap)	0.192	1.0000
	(I-Jn) - (II-Jn)	-0.000234	1.0000
	(I-Jn) - (II-My)	-0.876	0.9803
	(I-My) - (II-Ap)	-0.00772	1.0000
	(I-My) - (II-Jn)	-0.192	1.0000
	(I-My) - (II-My)	-0.201	1.0000
	(II-Ap) - (II-Jn)	-0.192	1.0000
	(II-Ap) - (II-My)	-0.201	1.0000
	(II-Jn) - (II-My)	-0.875	0.9803
Eva	(I-Jl) - (I-Jn)	0.194	0.9946
	(I-Jl) - (I-My)	1.22	0.1037
	(I-Jl) - (II-Ap)	0.212	1.0000
	(I-Jl) - (II-Jn)	0.755	0.4925
	(I-Jl) - (II-My)	0.885	0.3611
	(I-Jn) - (I-My)	1.03	0.2833
	(I-Jn) - (II-Ap)	0.210	1.0000
	(I-Jn) - (II-Jn)	0.561	0.8046
	(I-Jn) - (II-My)	0.690	0.6708
	(I-My) - (II-Ap)	0.200	1.0000
	(I-My) - (II-Jn)	-0.468	0.9545
	(I-My) - (II-My)	-0.339	0.9905
	(II-Ap) - (II-Jn)	-0.205	1.0000
	(II-Ap) - (II-My)	-0.204	1.0000
	(II-Jn) - (II-My)	0.130	0.9999
VespaCatch	(I-Jl) - (I-Jn)	-0.629	0.3254
	(I-Jl) - (I-My)	0.470	0.8534
	(I-Jl) - (II-Ap)	1.67	0.0836
	(I-Jl) - (II-Jn)	-0.405	0.8091
	(I-Jl) - (II-My)	-0.000004	1.0000
	(I-Jn) - (I-My)	1.10	**0.0316**
	(I-Jn) - (II-Ap)	2.30	**0.0020**
	(I-Jn) - (II-Jn)	0.223	0.9650
	(I-Jn) - (II-My)	0.629	0.3254
	(I-My) - (II-Ap)	1.20	0.4476
	(I-My) - (II-Jn)	-0.875	0.1841
	(I-My) - (II-My)	-0.470	0.8534
	(II-Ap) - (II-Jn)	-2.08	**0.0090**
	(II-Ap) - (II-My)	-1.67	0.0836
	(II-Jn) - (II-My)	0.405	0.8091
Econex	(I-Jl) - (I-Jn)	0.0445	1.0000
	(I-Jl) - (I-My)	0.571	0.5696

	(I-Jl) - (II-Ap)	1.75	**0.0158**
	(I-Jl) - (II-Jn)	0.302	0.9349
	(I-Jl) - (II-My)	0.363	0.8758
	(I-Jn) - (I-My)	0.526	0.6624
	(I-Jn) - (II-Ap)	1.70	**0.0213**
	(I-Jn) - (II-Jn)	0.258	0.9680
	(I-Jn) - (II-My)	0318	0.9279
	(I-My) - (II-Ap)	1.18	0.3084
	(I-My) - (II-Jn)	-0.268	0.9786
	(I-My) - (II-My)	-0.208	0.9937
	(II-Ap) - (II-Jn)	-1.45	0.0967
	(II-Ap) - (II-My)	-1.39	0.1304
	(II-Jn) - (II-My)	0.0606	1.0000

Figure S1. Selectiveness of the four types of baited traps tested throughout the study. Bars represent the Mean and the whiskers are Standard deviations. The tested traps were abbreviated as VespaCatch® trap (V), Econex® trap (X), Eva® trap (E) and prototype (R).

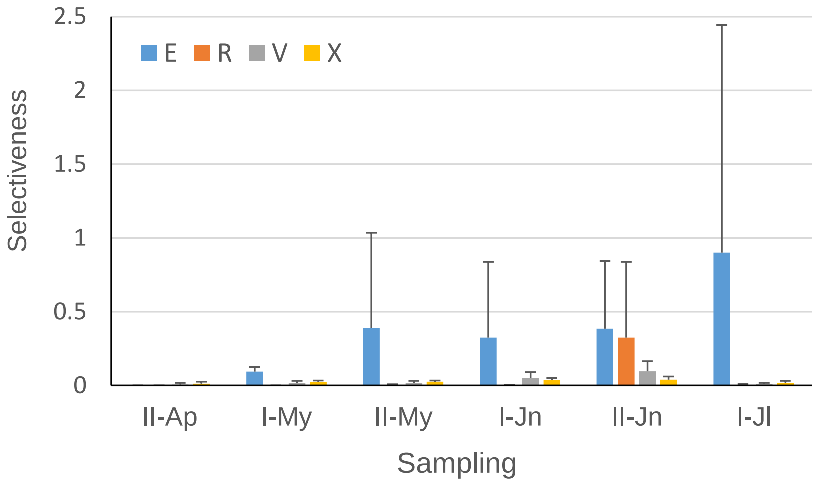

Table S3. Post-hoc tests for Selectiveness. Least-squares means for the fixed factor **Trap** of the GLMM model: **Selectiveness** (log-transformed) ~ Trap * Sampling + (1 ǀ trap_ID). Pairwise significantly differences ($p \leq 0.05$) in **bold**. The tested traps were abbreviated as VespaCatch® trap (V), Econex® trap (X), Eva® trap (E) and prototype (R).

Sampling	Traps	Estimate	p-value
II-April	R - E	0.000	1.0000
	R - V	-1.039	0.2640
	R - X	-1.794	**0.0107**
	E - V	-1.039	0.2640
	E - X	-1.794	**0.0107**
	V - X	-0.755	0.5457
I-May	R - E	-4.531	**< 0.0001**
	R - V	-2.335	**0.0009**
	R - X	-2.922	**< 0.0001**
	E - V	2.196	**0.0010**
	E - X	1.609	**0.0274**
	V - X	-0.587	0.7295
II-My	R - E	-3.788	**< 0.0001**

	R - V	-2.554	**0.0002**
	R - X	-2.490	**0.0003**
	E - V	1.234	0.1675
	E - X	1.298	0.1343
	V - X	0.064	0.9995
I-June	R - E	-3.495	**< 0.0001**
	R - V	-3.313	**< 0.0001**
	R - X	-3.178	**< 0.0001**
	E - V	0.181	0.9901
	E - X	0.316	0.9511
	V - X	0.135	0.9952
II-June	R - E	-4.464	**< 0.0001**
	R - V	-3.867	**< 0.0001**
	R - X	-2.931	**< 0.0001**
	E - V	0.597	0.7474
	E - X	1.532	0.0542
	V - X	0.935	0.3558
I-July	R - E	-4.417	**< 0.0001**
	R - V	-1.572	**0.0327**
	R - X	-1.952	**0.0045**
	E - V	2.845	**< 0.0001**
	E - X	2.465	**0.0004**
	V - X	-0.380	0.9079

Table S4. Post-hoc tests for the capture rate of Lepidoptera. Least-squares means for the fixed factor **Trap** of the GLMM model: **Capture rate of Lepidoptera** ~ Trap * Sampling + (1 | trap_ID). Pair-wise significantly differences ($p \leq 0.05$) in **bold**. The tested traps were abbreviated as VespaCatch® trap (V), Econex® trap (X), Eva® trap (E) and prototype (R).

Sampling	Traps	Estimate	p-value
II-April	R - E	-0.000028	1.0000
	R - V	-0.166699	0.9954
	R - X	-0.000029	1.0000
	E - V	-0.166671	0.9954
	E - X	-0.000001	1.0000
	V - X	0.166670	0.9954
I-May	R - E	-0.333358	0.9699
	R - V	-1.333363	0.2823
	R - X	-1.333360	0.2823
	E - V	-1.000005	0.4980
	E - X	-1.000002	0.4980
	V - X	0.000003	1.0000
II-My	R - E	-1.008632	0.5639
	R - V	-3.000019	**0.0006**
	R - X	-6.500019	**< 0.0001**
	E - V	-1.991387	**0.0418**
	E - X	-5.491387	**< 0.0001**
	V - X	-3.500000	**< 0.0001**
I-June	R - E	0.010958	1.0000
	R - V	-1.833362	0.0538
	R - X	-3.166693	**0.0001**
	E - V	-1.844320	0.0687
	E - X	-3.177651	**0.0002**
	V - X	-1.333331	0.2445
II-June	R - E	-0.212297	0.9918

	R - V	-0.333361	0.9657
	R - X	-4.500029	**< 0.0001**
	E - V	-0.121065	0.9985
	E - X	-4.287732	**< 0.0001**
	V - X	-4.166667	**< 0.0001**
I-July	R - E	0.010958	1.0000
	R - V	-1.333364	0.2445
	R - X	-4.833354	**< 0.0001**
	E - V	-1.344322	0.2753
	E - X	-4.844312	**< 0.0001**
	V - X	-3.499990	**< 0.0001**

Table S5. Post-hoc tests for the capture rate of native Vespidae. Least-squares means for the fixed factor **Trap** of the GLMM model: **Capture rate of native Vespidae** ~ Trap * Sampling + (1 ∣ trap_ID). Pair-wise significantly differences ($p \leq 0.05$) in **bold**. The tested traps were abbreviated as: VespaCatch® trap (V), Econex® trap (X), Eva® trap (E) and prototype (R).

Sampling	Traps	Estimate	p-value
II-April	R - E	0.000005	1.0000
	R - V	0.000017	1.0000
	R - X	0.000014	1.0000
	E - V	0.000012	1.0000
	E - X	0.000009	1.0000
	V - X	-0.000004	1.0000
I-May	R - E	0.590625	0.8953
	R - V	-0.576024	0.9020
	R - X	-2.242687	**0.0423**
	E - V	-1.166649	0.4670
	E - X	-2.833312	**0.0033**
	V - X	-1.666663	0.1656
II-My	R - E	0.316673	0.9838
	R - V	0.090626	0.9995
	R - X	-4.576023	**< 0.001**
	E - V	-0.226047	0.9931
	E - X	-4.892696	**< 0.001**
	V - X	-4.666649	**< 0.001**
I-June	R - E	-1.860276	0.1248
	R - V	-0.499985	0.9241
	R - X	-2.333314	**0.0221**
	E - V	1.360291	0.3709
	E - X	-0.473037	0.9426
	V - X	-1.833329	0.1067
II-June	R - E	-0.014104	1.0000
	R - V	-0.833337	0.7260
	R - X	-2.000001	0.0658
	E - V	-0.819233	0.7631
	E - X	-1.985898	0.0894
	V - X	-1.166665	0.4670
I-July	R - E	0.173083	0.9969
	R - V	-3.833301	**< 0.001**
	R - X	-4.999996	**< 0.001**
	E - V	-4.006384	**< 0.001**
	E - X	-5.173079	**< 0.001**
	V - X	-1.166695	0.4670

Table S6. Post-hoc tests for the capture rate of Coleoptera. Least-squares means for the fixed factor **Trap** of the GLMM model: **Capture rate of Coleoptera** ~ Trap * Sampling + (1 | trap_ID). Pair-wise significantly differences ($p \leq 0.05$) in **bold**. The tested traps were abbreviated as: VespaCatch® trap (V), Econex® trap (X), Eva® trap (E) and prototype (R).

Sampling	Traps	Estimate	p-value
II-April	R - E	0.000005	1.0000
	R - V	0.000017	1.0000
	R - X	0.000014	1.0000
	E - V	0.000012	1.0000
	E - X	0.000009	1.0000
	V - X	-0.000004	1.0000
I-May	R - E	0.590625	0.8953
	R - V	-0.576024	0.9020
	R - X	-2.242687	**0.0423**
	E - V	-1.166649	0.4670
	E - X	-2.833312	**0.0033**
	V - X	-1.666663	0.1656
II-My	R - E	0.316673	0.9838
	R - V	0.090626	0.9995
	R - X	-4.576023	**< 0.001**
	E - V	-0.226047	0.9931
	E - X	-4.892696	**< 0.001**
	V - X	-4.666649	**< 0.001**
I-June	R - E	-1.860276	0.1248
	R - V	-0.499985	0.9241
	R - X	-2.333314	**0.0221**
	E - V	1.360291	0.3709
	E - X	-0.473037	0.9426
	V - X	-1.833329	0.1067
II-June	R - E	-0.014104	1.0000
	R - V	-0.833337	0.7260
	R - X	-2.000001	0.0658
	E - V	-0.819233	0.7631
	E - X	-1.985898	0.0894
	V - X	-1.166665	0.4670
I-July	R - E	0.173083	0.9969
	R - V	-3.833301	**< 0.001**
	R - X	-4.999996	**< 0.001**
	E - V	-4.006384	**< 0.001**
	E - X	-5.173079	**< 0.001**
	V - X	-1.166695	0.4670
